# Hair cortisol concentrations are associated with hippocampal subregional volumes in children

**DOI:** 10.1038/s41598-020-61131-x

**Published:** 2020-03-17

**Authors:** Attila Keresztes, Laurel Raffington, Andrew R. Bender, Katharina Bögl, Christine Heim, Yee Lee Shing

**Affiliations:** 1 Brain Imaging Centre, Research Centre for Natural Sciences, Budapest, Hungary; 2 Center for Lifespan Psychology, Max Planck Institute for Human Development, Berlin, Germany; 3 Faculty of Education and Psychology, Eötvös Loránd University, Budapest, Hungary; 4 Department of Psychology, University of Texas at Austin, Texas, USA; 5 Departments of Epidemiology and Biostatistics & Neurology and Ophthalmology, Michigan State University, Michigan, USA; 6 Humboldt-Universität zu Berlin, Berlin, Germany; 7 Charité – Universitätsmedizin Berlin, corporate member of Freie Universität Berlin, Humboldt-Universität zu Berlin, and Berlin Institute of Health, Institute of Medical Psychology, Berlin, Germany; 8 Department of Biobehavioral Health, Pennsylvania State University, University Park, PA USA; 9 Institute of Psychology, Goethe University Frankfurt, Frankfurt am Main, Germany

**Keywords:** Cognitive neuroscience, Cognitive neuroscience, Hippocampus, Hippocampus, Stress and resilience

## Abstract

The human hippocampus, a brain structure crucial for memory across the lifespan, is highly sensitive to adverse life events. Stress exposures during childhood have been linked to altered hippocampal structure and memory performance in adulthood. Animal studies suggest that these differences are in part driven by aberrant glucocorticoid secretion during development, with strongest effects on the CA3 region and the dentate gyrus (CA3-DG) of the hippocampus, alongside associated memory impairments. However, only few pediatric studies have examined glucocorticoid associations with hippocampal subfield volumes and their functional relevance. In 84 children (age range: 6–7 years), we assessed whether volumes of hippocampal subregions were related to cumulative glucocorticoid levels (hair cortisol), parenting stress, and performance on memory tasks known to engage the hippocampus. We found that higher hair cortisol levels were specifically related to lower CA3-DG volume. Parenting stress did not significantly correlate with hair cortisol, and there was no evidence to suggest that individual differences in hippocampal subregional volumes manifest in memory performance. Our results suggest that the CA3-DG may be the hippocampal region most closely associated with hair cortisol levels in childhood. Establishing causal pathways underlying this association and its relation to environmental stress and memory development necessitates longitudinal studies.

## Introduction

The hippocampus, a bilateral brain structure in the medial temporal lobes, is crucial for learning and memory in humans^1^. In particular, it supports recalling highly specific details of events in our lives^2,3^, constructing memories^4^, associative or relational binding of information, and extracting knowledge from repeated experiences through generalization^5,6^. Importantly, it is also one of the most plastic^7^ brain structures, and is vulnerable to a wide range of influences across the lifespan^8,9^. The hippocampus is also implicated in the regulation of the hypothalamic-pituitary-adrenal (HPA) axis, and some evidence suggests the CA3 and the dentate gyrus subregions play a crucial role in stress adaptation^10^.

Renewed interest in the developmental plasticity of the hippocampus has been fueled by recent findings from high-resolution magnetic resonance imaging (MRI) research. Together, the evidence reveals a heterochronous pattern of protracted maturation across hippocampal subregions that continues well into adolescence^11–15^ and contributes to memory development^16^. This may be contrasted with prior suggestions that human hippocampal maturation occurrs by approximately 6 years of age^17–19^. Although the exact mechanisms underlying such plasticity are unclear, post-mortem studies in infants and very young children^17,20^, as well as data from various animal models^21,22^ implicate synaptogenesis, dendritic branching, and neurogenesis in such developmental changes.

The hippocampus may also be highly vulnerable to adverse influences during development^9,23,24^. One primary source of such vulnerability is stress: various forms of chronic childhood stress including trauma, maltreatment, abuse, poverty, and stress-related mental diseases are associated with subsequently smaller hippocampal volume in adulthood^25–36^. However, the retrospective nature of measuring childhood stress exposure and hippocampal volume in adulthood limits the validity of causal inferences that can be drawn from these extant findings. Investigations of stress-related effects on the hippocampus *during* childhood are scarce^31,37–46^ and their results are mixed. Prior studies evaluating associations between hippocampal volume and history of stress-related psychiatric disorders, or neglect and maltreatment reported both positive^38,46^ and negative^42,45,47,48^ effects. Those few studies in healthy, non-institutionalized or maltreated children also reported both negative^47^ and positive^49^ associations between questionnaire-based measures of cumulative stress exposure and hippocampal volume. Moreover, the only two longitudinal studies reported either no change^37^ or a decrease in hippocampal volume over time in the stress group^41^. It is possible that reduced measurement reliability and specificity in those studies may have limited the valid measurement of hypothesized change.

Dysfunctions of the HPA axis regulating neuroendocrine responses to stress have been associated with alterations of hippocampal structure and function, although human evidence is limited^33,50,51^. Animal studies suggest the hippocampus is particularly sensitive to increased levels of specific stress hormones, such as mineralocorticoids and glucocorticoids due to its high density of receptors for these hormones^52–55^, and hippocampal vulnerability to glucocorticoids^56^. Studies with various mammal species, including primates, showed that moderate elevation of glucocorticoid concentrations in the hippocampus can lead to decreased neuronal survival rates following various adverse neurological events. Moreover, extremely high glucocorticoid concentrations can directly result in neuronal damage, as evidenced by reductions in cell numbers and dendritic arborization^54,55,57^, with glucocorticoid-induced dendritic atrophy in the CA3 observed in rodents^58^ suggested as a potential mechanism in humans^34^. These effects can, in addition, underlie observations of stress-induced decreases in neurogenesis^59^.

However, evidence on the relationships between stress, stress hormones, and maturation is mixed, suggesting a complex pattern of underlying associations^60^. For instance, stress does not always lead to changes in glucocorticoid levels^34^, and can instead – in some instances – be associated with *reductions* of glucocorticoid secretion in both animals and humans as measured in salivary and hair samples^43,61–65^. Importantly, this may also negatively affect neural maturation: low levels of glucocorticoids can negatively affect neurogenesis in interaction with BDNF^66^. Other studies report weak and inconsistent associations^61,67,68^ or null results^67^ on adversity and cortisol concentrations in children. A recent meta-analysis on the association between adversity and hair cortisol levels confirms that adversity can be associated with both higher and lower hair cortisol levels and that the small association is moderated by type and timing of adversity, and sample characteristics^69^. In addition, trauma may lead to increased intraindividual variance in glucocorticoid levels^62^. Thus, cortisol concentrations do not seem to function as straightforward biomarkers of chronic stress, although experimental studies clearly show that levels are stress-sensitive in children^43,70^.

In samples of human children that were not selected on the basis of institutionalization or trauma exposure, only three studies have evaluated the association between hippocampal volume and cortisol. Whereas two of these reported no association between salivary cortisol levels and total hippocampal volume^43,71^, Pagliaccio and colleagues^49^ found that a positive link between stressful life events and left hippocampal volume was mediated by salivary cortisol levels such that cortisol was negatively associated with both stressful life events and hippocampal volume.

Taken together, definitive conclusions from prior studies on stress-related differences in the hippocampus during childhood and its underlying mechanisms are limited. First, most studies investigated stress exposure retrospectively. Second, these studies have exclusively used salivary assays for measuring cortisol levels that are highly variable and influenced by various circadian and endocrine influences as well as many other factors (e.g., hydration, caloric intake). In contrast, newer techniques developed to extract glucocorticoids from human hair afford a more reliable estimation of glucocorticoid concentrations accumulated over longer periods of time^72,73^. Third, associations of stress and cortisol do not appear to be uniform across the hippocampus^71^. In particular, the cornu ammonis regions and the dentate gyrus, the subregions with highest density of glucocorticoid receptors, may exhibit the largest negative cortisol effects in both animals and humans^33,52,53,55,57^. However, regionally specific effects within the human hippocampus have either been ignored or measured using methods with limited validity, largely due to methodological constraints imposed by standard resolution MRI.

Thus, in the present study, we investigated the association of cumulative levels of glucocorticoid concentrations, hippocampal subregional structure, and memory performance in an age-homogenous sample of 6-7-year old children. We assessed glucocorticoid concentrations via hair samples, and measured hippocampal subfield volumes using high-resolution MRI. We focused on this age range as it provides a window on development when the hippocampus is still undergoing changes, but when high-quality hippocampal structural images can already be acquired for reliable segmentation. Furthermore, we investigated whether self-reported parenting stress is associated with children’s cumulative cortisol levels and hippocampal structure. Parenting stress has been linked to attenuated salivary diurnal cortisol secretion in their children, potentially reflecting the child’s stress reaction to parenting stress^64^. Thus, assessing parents’ perceived stress may allow an approximation of children’s experienced stress levels, a construct difficult to measure reliably^64^. In addition, as the hippocampus has a key role in episodic memory, we evaluated children’s performance on memory tasks known to depend on hippocampal function. Finally, we assessed children’s performance on memory tasks, known to assess global (encoding spatial layouts^74^), and regionally specific (pattern separation in the dentate gyrus and CA3^3,75^) hippocampal function.

Based on the available evidence from primates on the mechanisms of stress-induced cellular changes in the hippocampus, we hypothesized that glucocorticoid concentrations are either positively or negatively associated with hippocampal structure. Specifically, we expected the dentate gyrus and CA3 regions to be the most sensitive to glucocorticoid concentrations. In addition, we anticipated these associations to manifest in memory performance: positive associations between total hippocampal volume and spatial memory, and between regional volumes of the dentate gyrus and CA3 and pattern separation.

## Methods and Materials

### Participants

We recruited 147 children, aged six to seven years from six Berlin districts (M_age_ = 2624 days [7.19 years], SD_age_ = 167 days; 67 girls) as part of a larger longitudinal study investigating SES-related stress effects on brain and cognition. Detailed description of the full sample, including risk of poverty, parental education and employment, child ancestry and bilingualism, as well as family status is given elsewhere^43^. Ethics approval was obtained from the ‘Ethik-Kommission der Deutsche Gesellschaft für Psychologie’ (Ethics Committee of the German Psychological Society). All research reported was performed in accordance with relevant guidelines and regulations. Informed consent was obtained from legal guardians of all participants: Parents provided written informed consent and children verbal assent. A subsample of randomly selected children had a magnetic resonance imaging (MRI) session that also incorporated behavioral tests on the mnemonic similarity task (N = 84; M_age_ = 2659 days, SD_age_ = 147 days; 44 girls). Due to movement related artifacts, three participants had missing data for hippocampal subfields (one for all, one for left hemisphere only, and one for entorhinal cortex only). Due to various technical issues, mnemonic similarity task data was missing for 3 children, and due to a lack of consent to cut hair, hair cortisol data was missing for 12 participants with MRI data. Altogether, 64 children (M_age_ = 2667 days, SD_age_ = 142 days; 31 girls) had complete data, however all analyses reported include all 84 children undergoing the MRI session (i.e., we did not exclude any participant with missing data).

### Hippocampal subfield measures

We acquired high-resolution, partial field of view (FoV) volumes of the medial temporal lobe using a T_2_-weighted, proton density (PD)-weighted turbo spin echo sequence on a 3 T Siemens Magnetom TrioTim syngo MRI scanner with the following parameters: FoV: 206 mm; repetition time (TR): 6,500 ms; echo time (TE): 16 ms; number of slices: 30; voxel size: 0.4 mm × 0.4 mm × 2.0 mm, oblique to the coronal plane, perpendicular to the longitudinal axis of the right hippocampus to cover the full bilateral hippocampus.

To delineate regions within the hippocampus, we implemented a pipeline previously described in Bender *et al*.^76^ using the Automated Segmentation of Hippocampal Subfields (ASHS) software tool^77^ with a custom atlas also created using ASHS from manual segmentations with excellent reliability from earlier studies in our laboratory. This approach has been shown to be highly reliable and valid in identifying hippocampal subfield boundaries in a lifespan developmental sample, including 6–14 year-old children^76^. Instead of using a single-atlas with a one-size-fits-all approach as e.g., Freesurfer, ASHS uses multi-atlas method – integrating information from manual segmentations of multiple hippocampi – that provides greater anatomical precision on the individual level. Importantly, this ensures superior inherent validity of our subfield measurement approach as compared to other automated approached such as Freesurfer, which may produce less reliable estimates and generates less reliable labels for small anatomical regions.

**Figure 1:**
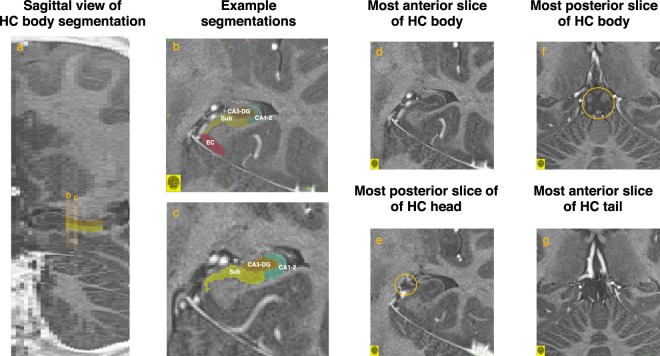
Illustration of Hippocampal Subfield Segmentations. (**a**) An example sagittal slice showing the extent of the hippocampal (HC) body segmentations were performed on. (**b**) The most anterior body slice with traces of all four regions overlaid. The four regions delineated comprised the Subiculum (Sub; with yellow color), a region including Cornu ammoni (CA) regions 1 and 2 (CA1-2; with turquoise color), a region including CA3 and the dentate gyrus (CA3-DG; with dark brown color), and the entorhinal cortex (EC; with red color). CA1-2, CA3-DG, and Subiculum were traced exclusively on HC body slices (from [D] to [F]), whereas EC was traced exclusively on (**b**) and 5 more slices anterior to it. (**c**) An additional example slice showing traces of CA1-2, CA3-DG, and Subiculum overlaid. (**d**) The most anterior HC body slice, defined as the first slice anterior to (**e**) which in turn was identified as the first slice on which the Uncus (circled) was clearly visible. (**f**) The most posterior slice of the HC body identified as the last slice on which the lamina quadrigemina (i.e., the inferior and superior colliculi; circled) are still clearly visible. (**g**) The first slice posterior to (**f**), where the lamina quadrigemina is not visible anymore. This slice was identified as belonging to the HC tail, and was not traced. Images created in ITK-SNAP^109^. See the Supplemental information for more details on hippocampal subfield volume assessment.

We delineated three regions within the hippocampal body (Fig. 1) bilaterally – the subiculum, a region including Cornu ammoni (CA) regions 1 and 2 (CA1-2), and a region including CA3 and the dentate gyrus (CA3-DG). We chose not to divide CA3 from dentate gyrus, but rather collapsed them into one subfield (CA3-DG) because methods to reliably and validly separate the two structures on images acquired with 3 Tesla scanners – even if high-resolution – are yet to be established^78^. For the same reason, we collapsed CA1 with CA2 into one subfield (CA1-2), and also collapsed presubiculum, subiculum, and parasubiculum into one subfield (Subiculum). These regions were delineated only on the body because validity of delineation of these subfields in the head or the tail of the hippocampus is still debated^78^. In addition to subfields within the hippocampal body, we delineated the entorhinal cortex (Fig. 1) on 6 consecutive slices anterior to the hippocampal body, starting with the most anterior slice of the hippocampal body. The manual demarcation protocol we had used to create the custom atlas which was used to guide the automatic segmentations by ASHS in this study are presented in detail in section 2.3.2 and Fig. 1. of a previous publication^76^. To account for differences in ROI volumes due to differences in head size, we used the analysis of covariance approach^79,80^ to correct volumetric estimates of subfields for intracranial volume (ICV). The adjusted volumetric data is used for all ROIs throughout the present report. (See the Supplemental information for more details on hippocampal subfield volume assessment.)

### Measurement of cortisol concentrations in hair samples

If children consented to hair sampling, a ~1 mm thick strand of hair was taken from as close to the skull as possible to the tip of the hair. Hair samples were then taped on aluminium foil, wrapped and shipped to the Dresden LabService GmbH, Germany for analysis. Cortisol concentrations were determined using liquid chromatography tandem mass spectrometry^72^. The analysis was constrained to the first 3 cm long segment of the hair from the skull, which – based on an estimated average of 1 cm / month hair growth^81^ – contained hormones secreted over 3 months prior to taking the hair sample^82^. Cortisol concentrations (pg/mg) were log-transformed for parametric statistics.

### Parenting stress measure

The Parenting Stress Inventory^83^ is a widely used questionnaire that assesses stress as a consequence of the parental role. Five subscales measure perceived stress due to child characteristics (Distractibility/Hyperactivity, Adaptability, Demandingness, Mood, Acceptability) and seven subscales measure parental characteristics and situational variables (Competence, Isolation, Attachment, Health, Role Restriction, Spouse/Parenting Partner Relationship, Depression). The validated German version of the questionnaire (Eltern-Belastungs-Inventar^84^) was completed by the parent who spent more time with the child and with the participating child in mind. Parents responded to 48 items on a 6-point scale ranging from strongly agree (0) to strongly disagree (5). The total score was divided by number of subscales (normally 12 subscales, but only 11 if the parent has no partner).

### Memory measures

Spatial memory and pattern separation integrity were tested using a grid memory task and a mnemonic similarity task, respectively. Both were computerized tasks performed on desktop computers with standard 21.5-inch screens. The grid memory task was based on^85^, and assessed memory for items and item-location associations. In brief, participants encoded locations of 15 sequentially presented (3 s) monochrome line drawings of everyday objects on gray-colored cells on a 6 × 6 grid. After a short delay, they performed a recognition task for the same pictures of studied objects randomly intermixed with 15 pictures of new objects. For each correctly recognized object, we asked them to point to the location of the given picture in the grid during encoding. As a measure of spatial memory, we used the percentage of correctly indicated locations for the 15 old items. For a detailed description of the task, see^43^.

The mnemonic similarity task was based on^86^, a continuous recognition memory task assessing participants’ ability to discriminate between memories of highly similar stimuli. Participants saw 162 pictures depicting everyday object, presented sequentially (trial duration: 4 s, interstimulus interval: 0.5 s). Critically, 48 pictures were repeated after a delay of either 2, 6, 10, or 14 intervening trials. Twenty-four of the pictures were repeated exactly, whereas 24 other pictures were repeated with a slightly different lure picture of an identical object. For each trial the children’s task was to identify pictures as “old” (exact repetition), “similar” (lure repetitions), or “new” (new items). Following^86^, we calculated a lure discrimination index as the proportion of “similar” responses to lure repetitions minus the proportion of “similar” responses to new items, that reflected participants’ ability to discriminate between highly similar memories. Prior to the task, participants completed training (via PowerPoint slides) to use the three response options with unlimited response times, and then proceeded to a practice version with timings identical to the experiment.

### Data conditioning and analysis

Data were screened for outliers, defined as having absolute values >3 SD from the mean. Outliers identified this way were further inspected and were subsequently excluded if obvious measurement errors were discovered. This resulted in the removal of one outlier (>10 SD above mean; apparent measurement error) from the hair cortisol data. Hair cortisol estimation was not successful in five children, as their values fell below detection limit, potentially reflecting measurement errors such as hair being cut too far from the skull^72^. No other outliers were removed. Thus, with the additional missing data for 12 participants due to lack of consent to cut hair, hair cortisol data was available for 66 of the 84 children.

Structural equation modeling (SEM) was used to investigate the relationship between hippocampal subfield volumes, hair cortisol measure, and parenting stress. Importantly, by creating a latent factor for each hippocampal subfield using the left and right volume measures, SEM provides independent estimates for each subfield factor free of measurement error and hemisphere specific variation. All models were computed using maximum likelihood estimation implemented in Onyx^87^ (version 1.0–1010). Standard goodness-of-fit indices, namely the root mean square error of approximation (RMSEA) and the comparative fit index (CFI), were used for evaluation of model fit. Models were considered a good fit with a RMSEA < 0.08 and CFI > 0.95 (e.g., Kline, 1998). The difference in χ^2^ fit statistics was used to compare nested models (Wald’s test), with the degrees of freedom being the difference in the number of free parameters. The threshold for statistical significance was *p* < 0.05. Both linear and quadratic associations were tested between variables of interest. In addition, to test for the robustness of results, we calculated 95% confidence intervals for all significant parameter estimates using 1000 bootstrapped resamples generated in lavaan^88^.

Age and Sex were included as covariates in further analyses, in light of earlier findings that have suggested an ongoing development of hippocampal structure in the age range examined^12,15,89,90^, and given known sex differences in HPA axis functioning ^e.g.,^ ^43^ as well as hippocampal subregions^14,89,91,92^. All indicator variables were converted to z-scores.

## Results

Table 1 provides the correlation matrix of all measures of interest plus Age. As can be seen, volumetric measures of hippocampal subfields were strongly intercorrelated, but subfield volumes and their sum – reflecting a total hippocampus (HC) body volume – were only weakly correlated with measures of hair cortisol concentrations, parenting stress, and memory performance, i.e., none of these weak correlations survive correction for multiple comparisons.

**Table 1:** Zero-order correlation matrix of all variables of interest.

	ERC left	ERC right	Sub left	Sub right	CA1-2 left	CA1-2 right	CA3-DG left	CA3-DG right	Total HC body	Hair cortisol	Parenting stress	Grid memory	LDI
ERC right	0.51***												
Sub left	0.37***	0.28**											
Sub right	0.27*	0.39***	0.6***										
CA1-2 left	0.34**	0.4***	0.54***	0.3**									
CA1-2 right	0.3**	0.49***	0.23*	0.52***	0.6***								
CA3-DG left	0.3**	0.46***	0.55***	0.36***	0.81***	0.61***							
CA3-DG right	0.25*	0.54***	0.25*	0.53***	0.54***	0.83***	0.68***						
Total HC body	0.4***	0.55***	0.71***	0.77***	0.76***	0.79***	0.83***	0.81***					
Hair cortisol	−0.13	−0.12	−0.05	−0.15	−0.2	−0.16	−0.23	−0.29*	−0.22				
Parenting stress	−0.25*	−0.15	−0.07	−0.09	0.00	−0.12	−0.09	−0.04	−0.1	0.09			
Grid memory	−0.01	−0.14	−0.04	0.05	−0.2	−0.15	−0.25*	−0.08	−0.12	−0.02	0.01		
LDI	0.06	0.16	0.14	0.1	0.14	0.03	0.16	0.05	0.13	−0.01	−0.03	0.15	
Age	−0.02	0.1	−0.08	0.02	0.14	0.22*	0.1	0.2	0.11	0.04	0.06	0.03	0.17

*Note*. ERC: entorhinal cortex, Sub: Subiculum, CA: Cornu Ammoni regions, DG: dentate gyrus, HC: hippocampus. Hair cortisol measures were log transformed. LDI: Lure discrimination index on the mnemonic similarity task. Age is age in days. *p < 0.05, **p < 0.01, ***p < 0.001, uncorrected for multiple comparisons.

### Associations between hippocampal subfield volumes and hair cortisol concentrations

**Figure 2:**
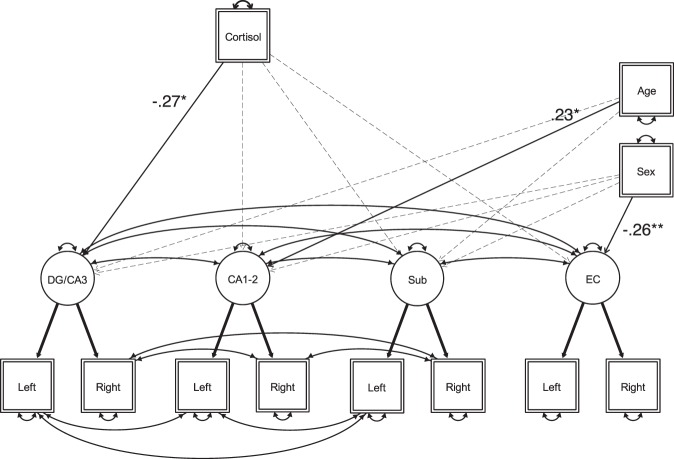
Schematic illustration of the hippocampal measurement model with regressions of hair cortisol concentrations (Cortisol), and covariates (Age and Sex) on hippocampal subfield volumes. DG/CA3: dentate gyrus–CA3, Sub: Subiculum: EC: Entorhinal cortex. Observed and latent variables are represented with rectangles and circles, respectively. Significant regression paths are shown as solid lines (with arrowhead) labeled with standardized parameter estimates. Non-significant paths are shown as dashed lines (with arrowhead). Estimated variances and covariances are also shown as solid lines. Thick solid lines represent path values fixed at 1. All parameter estimates are shown separately in Table 2, for better readability of the figure.

First, a latent factor of each hippocampal subfield was specified using the left and right volume measures (standardized for each side) of the corresponding region as observed indicators. The two factor loadings of each subfield indicator were fixed to 1. Latent factors had freely estimated covariances among each other, and residual variances of the indicators were not allowed to covary. The initial model showed a poor fit (χ^2^ = 131.33, df = 18, RMSEA = 0.208, CFI = 0.73). It is plausible that there were significant covariances among the residual variances of the indicators that were not specified. Because of their close anatomical relationship, this was particularly likely for hippocampal subregions in the same hemisphere. Therefore, we allowed the residual variances of subiculum, CA1-2, and CA3-DG within each hemisphere to be correlated. This led to a model with a good fit (χ^2^ = 14.66, df = 12, RMSEA = 0.039, CFI = 0.994), which is the measurement model (see bottom two rows of Fig. 2) that we used for all subsequent steps.

Next, hair cortisol concentration was entered as an observed predictor variable of the latent subfields, while controlling for Age and Sex. In this model (see Fig. 2, and Table 2 for parameter estimates of variances, covariances, path values, and test statistics), hair cortisol concentrations negatively predicted the volume of dentate gyrus (*p* = 0.019). Of our covariate measures, individual differences in Age accounted for a significant portion of the variance in CA1-2 volume (*p* = 0.032), and showed a trend for a positive association with CA3-DG (*p* = 0.065), whereas Sex accounted for a significant portion of the variance in EC volume (*p* = 0.005), reflecting that girls had larger EC than boys. No other paths predicting brain volume were significant. Confidence intervals calculated from bootstrapped samples (see Table S3), provided support for the robustness of these associations. There were no non-linear associations between hair cortisol and hippocampal volumes (all standardized *β*’s < 0.11 and all *p*’s > 0.13 for quadratic terms).

**Table 2:** Parameter estimates of the hippocampal measurement model, with regressions of hair cortisol, and covariates (age, sex) on hippocampal subfield volumes.

Model fit	**χ**^**2**^ = **31.26, df = 38, RMSEA = 0, CFI = 1**	
	Parameter estimates (Standard error)	Δχ^2^(1)
**Latent variable variances**		
DG/CA3	0.58 (0.12)	—
CA1-2	0.52 (0.12)	—
Sub	0.57 (0.12)	—
EC	0.43 (0.11)	—
**Latent variable covariances**		
DG/CA3 – CA1-2	0.49 (0.11)	—
DG/CA3 – Sub	0.27 (0.10)	—
DG/CA3 – EC	0.33 (0.09)	—
CA1-2 – Sub	0.24 (0.10)	—
CA1-2 – EC	0.32 (0.08)	—
Sub – EC	0.30 (0.09)	—
**Indicator covariances**		
***Left hippocampus***		
DG/CA3 – CA1-2	0.29 (0.09)	—
DG/CA3 – Sub	0.28 (0.08)	—
CA1-2 – Sub	0.30 (0.08)	—
***Right hippocampus***		
DG/CA3 – CA1-2	0.20 (0.07)	—
DG/CA3 – Sub	0.20 (0.07)	—
CA1-2 – Sub	0.23 (0.08)	—
**Regression paths**		
Hair cortisol onto DG/CA3	−0.27 (0.11)	5.47*
Hair cortisol onto CA1-2	−0.18 (0.11)	2.63
Hair cortisol onto Sub	−0.11 (0.12)	0.83
Hair cortisol onto EC	−0.10 (0.10)	0.97
Age onto DG/CA3	0.20 (0.11)	3.41
Age onto CA1-2	0.23 (0.10)	4.58*
Age onto Sub	−0.01 (0.11)	0.01
Age onto EC	0.90 (0.10)	0.71
Sex onto DG/CA3	−0.08 (0.10)	0.70
Sex onto CA1-2	−0.13 (0.10)	1.93
Sex onto Sub	−0.04 (0.10)	0.12
Sex onto EC	−0.26 (0.09)	7.77**

*Note*. Standardized parameter estimates shown with standard errors in parenthesis. Error variances are not shown. *p < 0.05, **p < 0.01, ***p < 0.001.

### Associations between parenting stress and hippocampal subfield volumes of children

Next, we explored associations between parental stress and children’ hippocampal subfield volumes (see Figure S1, and Table S1). We specified parenting stress as a manifest variable with a regression paths to each of the four hippocampal subregion factors in the measurement model. In this model, parenting stress of parents was found to negatively predict the volume of entorhinal cortex (*p* = 0.018). Covariances with Age and Sex provided a similar pattern to the one observed in the hippocampal–hair cortisol model described above (see Table S1). No other paths predicting brain volume were significant. Again, bootstrapping replicated the results (see Table S3). There were no non-linear associations between parenting stress and hippocampal volumes (all standardized *β*’s < 0.63 and all *p*’s > 0.15 for quadratic terms).

### Associations of hair cortisol concentrations, parenting stress, hippocampal subfield volumes and memory

A combined model with cortisol levels and parenting stress (see Figure S2 and Table S2) yielded an identical pattern of results with similar parameter estimates. Importantly, covariance between children’s hair cortisol and parenting stress was positive, but not significant (r = 0.08, *SE* = 0.09, *p* = 0.97). There were also no non-linear associations between children’s hair cortisol, parenting stress and hippocampal volumes (all standardized *β*’s < 0.11 and all *p*’s > 0.13 for quadratic terms).

Next, spatial memory performance and lure discrimination index were entered in this combined model as manifest dependent variables regressed on the latent hippocampal subfield variables. Covariance between spatial memory and the lure discrimination index was positive, but not significantly different from zero (r = 0.18, *SE* = 0.12, *p* = 0.14). Hippocampal subfield volumes covaried both positively and negatively with memory performance, but never significantly different from zero (all standardized *β*’s < 0.26, all *p*’s > 0.52). There were also no non-linear associations between hippocampal volumes and memory (all standardized *β*’s < 0.11 and all *p*’s > 0.12 for quadratic terms).

Finally, in an additional analysis, we ruled out the potential confounding effect of participant’s hair color on our main results. First, regressing hair cortisol on hair color revealed no significant association between the two (R^2^ = −0.014, F(3,87) = 0.58, p = 0.63, ns.). Second, when entering hair color as a dummy variable in a regression of right CA3-DG volume on hair cortisol, the significant association between hair cortisol and right CA3-DG volume remained significant (β_standardized_ = 0.30, p = 0.021).

## Discussion

In this study, we examined whether 6-to-7-year-old children’s hippocampal subregional volumes were associated with cumulative cortisol concentrations and parenting stress, as well as the functional relevance of differences in volume as indicated by memory performance. Based on the assumption that cumulative stress may be associated with altered cortisol concentrations, that in turn affect hippocampal volumes, and performance on hippocampal memory tests, we hypothesized a correlational relationship among these variables in our developmental sample.

Our most important finding is that hair cortisol concentrations over approximately three months preceding the study were negatively associated with the volume of the CA3-DG region of the hippocampus. Crucially, this may suggest that effects of cortisol accumulation may affect brain structure in healthy humans as early as 6 years of age. Such effects may have potential consequences for children in terms of learning and stress regulation. Given the cross-sectional nature of our results, it is also possible that children with smaller CA3-DG regions have subsequently higher cortisol secretion or a third variable causally accounts for their association. Importantly, this main finding replicates, with rigorous semi-automated hippocampal segmentation methods, recent findings of a concurrent study^93^. In contrast, previous studies exploring associations between cortisol and hippocampus in human children used different measures of salivary cortisol concentrations, and found mixed results ^e.g.,^ ^43^.

The specific association between cortisol and CA3-DG volume in children may reflect the increased vulnerability of this hippocampal region to high levels of cortisol concentrations via decreased survival rate of new neurons produced in the DG^54,55,57,59^, higher glucocorticoid-induced dendritic atrophy in the CA3^34^, as well as a higher concentration of glucocorticoid receptors in the DG and CA regions that renders these regions more vulnerable to the diverse adverse effects of cortisol on hippocampal network integrity^33,52,53,55,57^.

We also found a negative association between parenting stress and volume in an adjacent neocortical region. We can speculate that this association may reflect a lack of resources that may support extra-hippocampal cortical development at this age by providing environmental stimulation. For instance, parental stress may be associated with limited resources to select environmentally stimulating experiences for the child. Alternatively, children with smaller EC volumes may evoke more parenting stress in their parents, or smaller EC volumes could reflect a heritable susceptibility to higher stress perception. However, why this may be specific to EC volumes – and not total HC – is unclear. Future studies should explore whether this association replicates in other samples, preferably in bidirectional longitudinal designs.

In this study associations between memory performance and volumetric measures of hippocampal subfields were not significantly different from zero, as we hypothesized. Thus, it is not possible to draw conclusions on whether the association between cortisol levels and hippocampus manifests in worse memory performance in children. Note that in another study using the same sample we found a trend for a *negative* association between total hippocampal volume (estimated with Freesurfer) and memory^43^. The lack of a robust association between memory and hippocampal structure in this sample seems to contradict earlier reports finding significant associations between performance on various memory tasks and total or region-specific hippocampal volumes^11–14,89,92,94,95^. Most of these studies found a positive association between volume and memory (but see meta-analysis by Van Petten, 2004 that reported negative association). However, there is some evidence suggesting that the direction of the association may vary with age, across subregions, and along the longitudinal axis within the hippocampus^89,90^. These inconsistencies suggest a complicated relationship between memory and hippocampal volume that unfolds during development. For instance, Tamnes *et al*.^92^ found that cross-sectional associations (larger volume is better for retrieval) dissociate from longitudinal (decrease in volume is better for learning). This reflects a wider problem of false inferences from cross-sectional data with respect to true developmental patterns^96^, see also^97^. Another recent study^89^ found that the direction of the association between the volume of the CA1 region in the hippocampal head and memory shifts around the age of 6 from positive to negative. Thus, associations between memory and hippocampal volume may be better captured in larger cross-sectional samples spanning wider age ranges, or even better, by longitudinal sampling. Given the above considerations, the narrow age range of 6–7 years may have hindered the detection of any association of memory with hippocampal volume. This is especially so as pattern separation has been shown to develop beyond this age^12,98^. The possibility of an age range around or below the age of 6, during which hippocampal subfield volume is negatively associated with memory performance ^cf.^ ^89^ leads to the intriguing possibility that a smaller volume associated with higher cortisol levels may be beneficial for children of this age. This idea needs further exploration.

Another possibility is that the limited statistical power given the sample size and nature of the tasks in our study precluded detection of associations between memory and hippocampal volume. For instance, the continuous recognition task version of the mnemonic similarity task^86^ may put higher demands on working memory than the incidental task version^99^ used in our previous study^12^ where an association between age-related differences in hippocampal subfield volumes and memory was found. This is because in a continuous recognition task, participants are encouraged to monitor the stream of stimuli for reoccurring items and update their memories whenever an item appears again (as that item will no longer reoccur), which is very similar to the task demands of an *n*-back task. Thus, performance on this task may also heavily depend on prefrontal regions supporting working memory^100^, which may mask any association to the hippocampus present. Given the widely demonstrated association between hippocampal structure and memory performance, future studies may benefit from using child-adapted versions of tests of hippocampal function such as statistical inference^14^, holistic recognition^101^, and associative binding^102^.

We did not find a significant association between parenting stress (of the parents) and hair cortisol concentrations (in the children). This may be due to the fact that parenting stress is an indirect, and potentially unreliable approximation of children’s stress levels, as well as a lack of statistical power, or a true lack of the association (either between parenting stress and children’s stress, or children’s stress and their hair cortisol levels). In addition, our parenting stress questionnaire did not specify that parents should fill it with respect to the preceding 3 months. Therefore, the time window covered by the parenting stress questionnaire did not precisely overlap with the three-month time window covered by the hair cortisol measurement. Self-reported parenting stress may also be a poor approximation of the parents’ actual stress level^103^. However, a recent meta-analysis^69^ suggests that the association of adversity and hair cortisol levels is either positive and small in effects size or negative and moderate. Thus, our study may simply be underpowered to detect small-to-moderate associations. In addition, the non-significant association between hair cortisol in children and parenting stress of the parents may indicate that the parenting stress perceived by the parents does not translate directly into children’s experienced stress ^e.g.,^ ^64^. Alternatively, there may not be a reliable association of hair cortisol levels and stress exposure in children who were not institutionalized or maltreated ^e.g.,^ ^67,68^. Overall, this is in line with the notion that stress and cortisol have a complex, and yet poorly understood relationship^60^. Even in adults, and humans in general, within-person measurement of hair cortisol and its coupling with self-reported stress remains to be robustly corroborated (for evidence in primates, see^73^, for evidence in children see^104^, for a review see^105^). Our results do not provide evidence for the notion that the link between hair cortisol and hippocampal structure in children derives through a stress mechanism. Nevertheless, measurement of hair cortisol may be a promising tool for developmentalists investigating the relationship of cumulative cortisol concentrations (whether stress derived or not) and brain structure. This study provides initial evidence for a link between neural development and cumulative cortisol levels.

Finally, the negative association between CA3-DG and hair cortisol in children should be interpreted with caution until further variables beyond hair color can be ruled out as potential confounds. For instance, there is some evidence that hair washing, or repeated exposure to water (e.g., regular swimming) can leach cortisol out of primate^106^ and human hair^107^, although human evidence is mixed and suggests that proximal segments of hair are not affected^107,108^. Importantly, a concurrent study similarly finding a negative CA3-DG – hair cortisol association, has successfully ruled out the modifying effect of hair washing^93^.

Based on the limitations touched upon in the above sections, future longitudinal studies may shed light on a true developmental lead-lag relationship between hair cortisol and hippocampal volume, or rather provide support for a common – perhaps genetic – mechanism. Longitudinal assessments are also needed to uncover a potential cascading effect of stress hormones. For instance, it is possible that effects on memory performance manifest only after longer periods of exposure to either too high or too low levels of cortisol concentrations. The development of valid assessment tools for measuring stress in children aged 6–7 years may further contribute to understanding the mechanisms underlying the observed association between hair cortisol and CA3-DG.

In sum, this study established a link between the accumulation of the stress hormone cortisol over a period of approximately three months and volume of the hippocampal region CA3-DG. It remains to be clarified to what extent our finding derives from environmental stress exposure, or mechanisms other than stress, such as genetic factors or toxin exposure, and whether the hair cortisol – CA3-DG association translates to effects on memory performance. We believe that uncovering these mechanisms could effectively guide efforts to understand developmental psychopathologies related to interactions of hormonal stress regulation in childhood and neurocognitive development.

## Supplementary information

Supplemental Information.
