# Supplementary material for: Hair cortisol concentrations are associated with hippocampal subregional volumes in children
Source: Sci Rep. 2020 Mar 17;10:4865. doi: 10.1038/s41598-020-61131-x (PMC7078215; doi:10.1038/s41598-020-61131-x)
Supplement: Supplementary file 1 — Supplemental Information. [file 41598_2020_61131_MOESM1_ESM.pdf]

Supplementary Information for

Hair cortisol concentrations are associated with hippocampal subregional volumes in children

Attila Keresztes[1,2,3*], Laurel Raffington[2,4], Andrew R. Bender[2,5], Katharina Bögl[6], Christine Heim[7,8,†], Yee Lee Shing[2,9,†]

[1]Brain Imaging Centre, Research Centre for Natural Sciences, Budapest, Hungary

[2]Center for Lifespan Psychology, Max Planck Institute for Human Development, Berlin, Germany

[3]Faculty of Education and Psychology, Eötvös Loránd University, Budapest, Hungary

[4]Population Research Center, University of Texas at Austin, Texas, USA

[5]Departments of Epidemiology and Biostatistics & Neurology and Ophthalmology, Michigan State University, Michigan, USA

[6]Humboldt-Universität zu Berlin, Berlin, Germany

[7]Charité – Universitätsmedizin Berlin, corporate member of Freie Universität Berlin, Humboldt-Universität zu Berlin, and Berlin Institute of Health, Insitute of Medical Psychology, Berlin, Germany

[8]Department of Biobehavioral Health, Pennsylvania State University, University Park, PA, USA

[9]Institute of Psychology, Goethe University Frankfurt, Frankfurt am Main, Germany

† Shared last authorship

Methods and Materials for hippocampal subfield measures

Magnetic resonance imaging (MRI) acquisition

We acquired high-resolution, partial field of view (FoV) volumes of the medial temporal lobe using a T_2-weighted, proton density (PD)-weighted turbo spin echo sequence on a 3 T Siemens Magnetom TrioTim syngo MRI scanner with the following parameters: FoV: 206 mm; repetition time (TR): 6,500 ms; echo time (TE): 16 ms; number of slices: 30; voxel size: 0.4 mm × 0.4 mm × 2.0 mm, oblique to the coronal plane, perpendicular to the longitudinal axis of the right hippocampus to cover the full bilateral hippocampus. Scanner operators were trained to visualize the hippocampal body and a key landmark used in tracing of the hippocampus (i.e., the Stratum Radiatum, Lacunosum, Moleculare (SRLM) layer within the hippocampal body) and inspected the PD images immediately after the PD sequence was run. If the hippocampal body and the SRLM were not visualized at least on 6 consecutive slices, the PD sequence was repeated until a maximum of 2 times.

Training children for the MRI session

Prior to scanning, participants were given detailed instruction on why and how to stay still in the MRI. For examples they were shown "sharp" and "vague" images of brain scans, and were shown what type of distortions movements result in. All participants underwent a mock scanning session in order to train them to stay as still as possible during scanning. During scanning, they were watching videos.

Delineating subfields

To delineate regions within the hippocampus, we used a pipeline previously described in Bender et al. [1]. Briefly, hippocampal subfields were segmented using the Automated

Segmentation of Hippocampal Subfields (ASHS) software tool [2] using a custom atlas also created using ASHS from manual segmentations with excellent reliability from earlier studies in our laboratory. This approach has been shown to be highly reliable and valid in identifying hippocampal subfield boundaries [1].

Defining hippocampal body ranges

The range of slices containing the hippocampal body was defined by K.B. and A.K., using landmarks described in detail in Bender et al. [1]. Briefly, the first slice in the anterior-posterior direction was defined as the slice where the uncus or tissue belonging to the hippocampal head was not visualized anymore, whereas the last slice was defined as the last slice where any colliculus of the lamina quadrigemina was still visualized. To establish reliability of identification of the boundaries of the hippocampal body, K.B. and A.K., an experienced manual tracer, began by ranging the hippocampal body on blocks of 12 cases of the sample. The stopping rule was to declare inter-rater reliability if a Cohen's kappa > .75 would be reached, separately for starting and ending slices bilaterally. Cohen' kappa is a measure of inter-rater reliability for ratings on nominal scales [3], with values > .75 indicating substantial reliability. After each 12 cases, K.B. and A.K. discussed discrepancies, obvious errors were corrected, an agreement by Cohen's kappa was calculated on the cumulative sample of sets of 12 cases, and then the two agreed on discrepant decisions to be used for further analyses. The target Cohen's kappa was reached after the 4th attempt (including 48 PD images; starting left: .883, starting right: .820, ending left: .878, ending right: .910), indicating excellent reliability. K.B. tested intra-rater reliability of her own rangings on the first 13 cases after a delay of 1 year. This test provided excellent reliability (Cohen's kappa's for starting left: .803, starting right: .874, ending left: .885, ending right: .766). After both intra- and inter-rater reliability was established, only K.B ranged the remainder of the sample.

Manual correction of segmentations

Automatic segmentation outputs from ASHS were visually inspected by K.B. and A.K., and A.K. corrected segmentations manually for small errors using ITK-SNAP [4]. For three cases, errors called for complete manual delineation of the target regions. These manual tracings were performed by A.K. using methods detailed in Bender et al., [1] and Keresztes et al. [5].

Adjustments of hippocampal volumes by intracranial volume

Finally, to account for differences in ROI volumes due to differences in head size, we used the analysis of covariance approach [6,7] to correct volumetric estimates of subfields for intracranial volume (ICV). The adjusted volumetric data is used for all ROIs throughout the present report. As this correction uses the regression slope of each subfield volume on ICV, we tested for the significance of a Sex × ICV interaction. This interaction was significant for the volume of left entorhinal cortex. Thus, only for this region, we calculated sex specific regression slopes between the subfield and ICV. The ICV estimates were obtained using the brain extraction tool in FSL 5.0 [8] using procedures described in Bender et al. [9].

Table S1. Parameter estimates of the hippocampal measurement model, with regressions of parenting stress, and covariates (Age and Sex) on hippocampal subfield volumes

Model fit	**χ^2 = 33.98, df = 38, RMSEA = 0, CFI = 1**	
	Parameter estimates (Standard error)	$\Delta\chi^2(1)$
**Latent variable variances**		
DG/CA3	0.92 (0.13)	–
CA1-2	0.87 (0.12)	–
Sub	0.98 (0.12)	–
EC	0.74 (0.11)	–
**Latent variable covariances**		
DG/CA3 – CA1-2	0.80 (0.11)	–
DG/CA3 – Sub	0.46 (0.10)	–
DG/CA3 – EC	0.55 (0.09)	–
CA1-2 – Sub	0.41 (0.10)	–
CA1-2 – EC	0.55 (0.08)	–
Sub – EC	0.53 (0.08)	–
**Indicator covariances**		
Left hippocampus		
DG/CA3 – CA1-2	0.27 (0.08)	–
DG/CA3 – Sub	0.27 (0.08)	–
CA1-2 – Sub	0.29 (0.08)	–
Right hippocampus		
DG/CA3 – CA1-2	0.21 (0.07)	–
DG/CA3 – Sub	0.21 (0.07)	–
CA1-2 – Sub	0.24 (0.08)	–
**Regression paths**		
Parenting stress onto DG/CA3	-0.09 (0.10)	0.57
Parenting stress onto CA1-2	-0.10 (0.10)	0.68
Parenting stress onto Sub	-0.11 (0.10)	0.74
Parenting stress onto EC	-0.30 (0.09)	5.64*
Age onto DG/CA3	0.22 (0.11)	2.75
Age onto CA1-2	0.28 (0.11)	4.16*
Age onto Sub	-0.02 (0.11)	0.03
Age onto EC	0.11 (0.10)	0.72
Sex onto DG/CA3	-0.14 (0.10)	1.47
Sex onto CA1-2	-0.20 (0.10)	2.79
Sex onto Sub	-0.07 (0.10)	0.26
Sex onto EC	-0.39 (0.09)	9.79**

Note. Standardized parameter estimates shown with standard errors in parentheses. Error variances are not shown. *: $p < 0.05$, **: $p < 0.01$, ***:$p < 0.001$

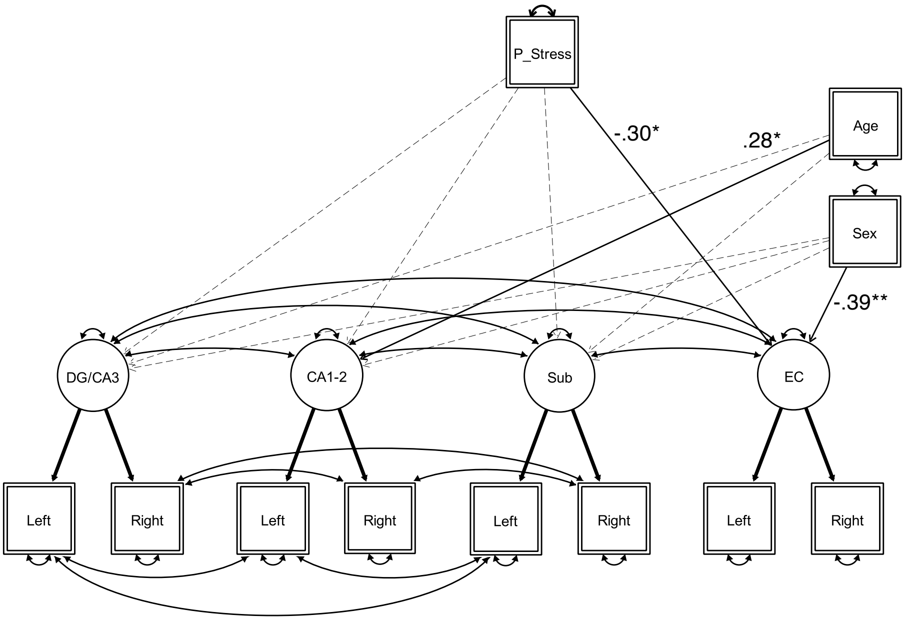

Figure S1. Schematic illustration of the hippocampal measurement model with regressions of parenting stress of the parents (P_Stress), and covariates (Age and Sex) on hippocampal subfield volumes. DG/CA3: dentate gyrus–CA3, Sub: Subiculum: EC: Entorhinal cortex. Observed and latent variables are represented with rectangles and circles, respectively. Significant regression paths are shown as solid lines labeled with standardized parameter estimates. Non-significant paths are shown as dashed lines. Estimated variances and covariances are also shown as solid lines. Thick solid lines represent path values fixed at 1. All parameter estimates are shown separately in Table S1, for better readability of the figure.

Table S2. Parameter estimates of the hippocampal measurement model, with regressions of hair cortisol, parenting stress, and covariates (Age and Sex) on subfield volumes

Model fit	**χ^2 = 38.96, df = 45, RMSEA = 0, CFI = 1**	
	Parameter estimates (Standard error)	$\Delta\chi^2(1)$
**Latent variable variances**		
DG/CA3	0.82 (0.12)	–
CA1-2	0.82 (0.12)	–
Sub	0.97 (0.12)	–
EC	0.73 (0.11)	–
**Latent variable covariances**		
DG/CA3 – CA1-2	0.73 (0.11)	–
DG/CA3 – Sub	0.42 (0.1)	–
DG/CA3 – EC	0.51 (0.09)	–
CA1-2 – Sub	0.39 (0.09)	–
CA1-2 – EC	0.52 (0.08)	–
Sub – EC	0.51 (0.08)	–
**Indicator covariances**		
Left hippocampus		
DG/CA3 – CA1-2	0.27 (0.08)	–
DG/CA3 – Sub	0.28 (0.08)	–
CA1-2 – Sub	0.29 (0.08)	–
Right hippocampus		
DG/CA3 – CA1-2	0.21 (0.07)	–
DG/CA3 – Sub	0.20 (0.07)	–
CA1-2 – Sub	0.24 (0.08)	–
Hair cortisol – Parenting stress	0.08 (0.09)	–
**Regression paths**		
Hair cortisol onto DG/CA3	-0.32 (0.11)	5.31*
Hair cortisol onto CA1-2	-0.22 (0.11)	3
Hair cortisol onto Sub	-0.13 (0.12)	0.71
Hair cortisol onto EC	-0.14 (0.1)	0.93
Parenting stress onto DG/CA3	-0.08 (0.1)	0.5
Parenting stress onto CA1-2	-0.1 (0.1)	0.64
Parenting stress onto Sub	-0.11 (0.1)	0.68
Parenting stress onto EC	-0.3 (0.09)	5.61*
Age onto DG/CA3	0.24 (0.11)	3.5
Age onto CA1-2	0.29 (0.1)	4.69*
Age onto Sub	-0.01 (0.1)	0.01
Age onto EC	0.12 (0.1)	0.83
Sex onto DG/CA3	-0.1 (0.1)	0.74
Sex onto CA1-2	-0.17 (0.1)	2
Sex onto Sub	-0.05 (0.1)	0.16
Sex onto EC	-0.38 (0.09)	8.8**

Note. Standardized parameter estimates shown with standard errors in parenthesis. Error variances are not shown. *: $p < 0.05$, **: $p < 0.01$

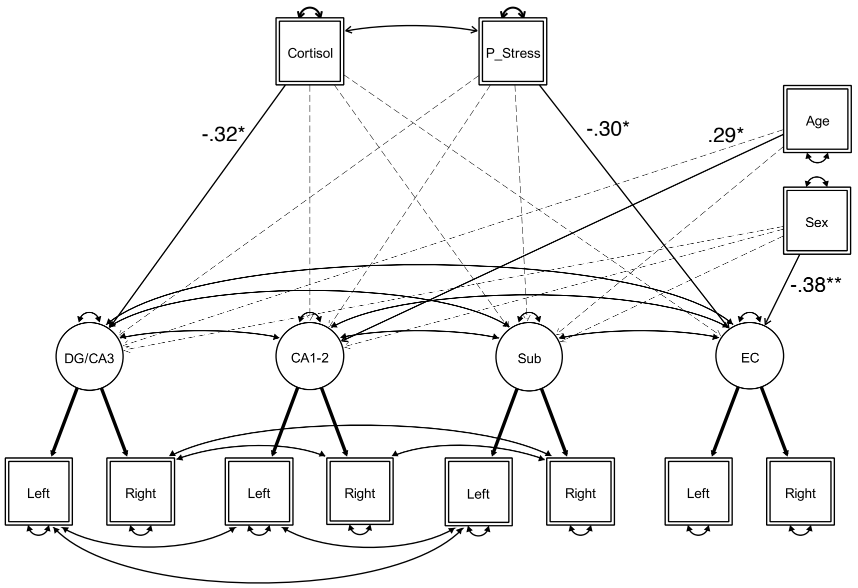

Figure S2. Schematic illustration of the hippocampal measurement model with regressions of hair cortisol concentrations (Cortisol), parenting stress of the parents (P_Stress), and covariates (Age and Sex) on hippocampal subfield volumes. DG/CA3: dentate gyrus–CA3, Sub: Subiculum: EC: Entorhinal cortex. Observed and latent variables are represented with rectangles and circles, respectively. Significant regression paths are shown as solid lines labeled with standardized parameter estimates. Non-significant paths are shown as dashed lines. Estimated variances and covariances are also shown as solid lines. Thick solid lines represent path values fixed at 1. All parameter estimates are shown separately in Table S2, for better readability of the figure.

Table S3. Mean and confidence intervals of 1000 bootstrapped resamples of significant parameter estimates

	Bootstrapped PE	95% CI
**HC CFA with hair cortisol**		
(Fig. 1, Table 1)		
Hair cortisol onto DG/CA3	-0.27	[-0.51,-0.05]
Age onto CA1-2	0.23	[0.01,0,45]
Sex onto EC	-0.26	[-0.45,-0.07]
**HC CFA with parenting stress**		
(Fig. S1, Table S1)		
Parenting stress onto EC	-0.21	[-0.40,-0.02]
Age onto CA1-2	0.22	[0.02,0.44]
Sex onto EC	-0.29	[-0.47,-0.11]
**HC CFA with hair cortisol and parenting stress**		
(Figure S2, Table S2)		
Hair cortisol onto DG/CA3	-0.27	[-0.52,-0.03]
Parenting stress onto EC	-0.22	[-0.40,-0.02]
Age onto CA1-2	0.23	[0.01,0.46]
Sex onto EC	-0.28	[-0.46,-0.09]

Note. PE: parameter estimate, CI: confidence interval, HC: Hippocampus, CFA: confirmatory factor analysis

References for Supplemental Information

1. Bender, A. R. *et al.* Optimization and validation of automated hippocampal subfield segmentation across the lifespan. *Hum. Brain Mapp.* **39**, 916–931 (2018).
2. Yushkevich, P. A. *et al.* Automated volumetry and regional thickness analysis of hippocampal subfields and medial temporal cortical structures in mild cognitive impairment. *Hum Brain Mapp* **36**, 258–287 (2015).
3. Cohen, J. A coefficient of agreement for nominal scales. *Educational and psychological measurement* **20**, 37–46 (1960).
4. Yushkevich, P. A. *et al.* User-guided 3D active contour segmentation of anatomical structures: significantly improved efficiency and reliability. *Neuroimage* **31**, 1116–1128 (2006).
5. Keresztes, A. *et al.* Hippocampal maturity promotes memory distinctiveness in childhood and adolescence. *PNAS* **114**, 9212–9217 (2017).
6. Jack, C. R. *et al.* Anterior temporal lobes and hippocampal formations: normative volumetric measurements from MR images in young adults. *Radiology* **172**, 549–554 (1989).
7. Raz, N. *et al.* Regional brain changes in aging healthy adults: general trends, individual differences and modifiers. *Cerebral cortex* **15**, 1676–1689 (2005).
8. Smith, S. M. Fast robust automated brain extraction. *Human brain mapping* **17**, 143–155 (2002).
9. Bender, A. R., Daugherty, A. M. & Raz, N. Vascular risk moderates associations between hippocampal subfield volumes and memory. *Journal of cognitive neuroscience* **25**, 1851–1862 (2013).
